# Thoughts on Chronic Inversion of the Uterus

**Published:** 1871-12

**Authors:** 


*Thoughts on Chronic Inversion of the Uterus.* By Henry Miller, M. D.

This pamphlet contains a very able article on a very interesting subject, and plainly shows that the author has thoroughly investigated the various plans of treatment advocated by different authors, for this condition of the uterus. He first speaks very favorably of the amputation of the organ, and shows, that the operation in successful cases does not in the least impair the health of the patient, but may in many respects promote the same. After this, the author says: " It had been settled as a cannon in obstetric medicine, that an inverted uterus cannot be reduced by any kind of manipulation, when any considerable time has elapsed since the displacement occurred. Few were bold enough to make the attempt, after the lapse of a week or two; but after the lapse of months, when involution had restored the organ to its unimpregnated size, none had the hardihood to venture upon such an experiment.* The profession were aroused in this country from servile submission to authority, by the publication of a case of chronic inversion of the uterus of six months duration, successfully reduced by Prof. James P. White, of Buffalo."

Our author speaks of the methods of manipulation practiced by Prof. White, first in this country and by Tyler Smith about the same time, in England, with great favor, but gives statistics showing more favorably for amputation in its results than restoration as thus far reported.

It seems to us, that after all, his statistics if properly interpreted will convince unbiased minds that re-position is more safe than amputation, and in all respects more satisfactory to the parties interested in such a procedure.

